# Clinical and laboratory characteristics of children under five hospitalized with diarrhea and bacteremia

**DOI:** 10.1371/journal.pone.0243128

**Published:** 2020-12-02

**Authors:** K. M. Shahunja, Tahmeed Ahmed, Md. Iqbal Hossain, Md. Munirul Islam, Mahmuda Begum Monjory, Abu Sadat Mohammad Sayeem Bin Shahid, Abu Syed Golam Faruque, Mohammod Jobayer Chisti

**Affiliations:** International Centre for Diarrhoeal Disease Research, Dhaka, Bangladesh; University of Georgia, UNITED STATES

## Abstract

**Background:**

Diarrhea is one of the leading causes of mortality in children under five globally. When it is associated with bacteremia, mortality is even higher. However, bacteraemia in diarrheal children has gained little attention in spite of its deleterious impact in under-five mortality. So, we aimed to evaluate associated clinical and laboratory factors for death in under-five children hospitalized with both diarrhea and bacteremia.

**Methods:**

In this retrospective cross-sectional study, we used patients’ electronic database of Dhaka Hospital of ‘icddr,b’, and enrolled all under-five children with diarrhea and bacterial growth in their blood samples on admission between June-2014 and May-2017. Clinical and laboratory characteristics were compared between those who died and who survived with a special attention to bacterial pathogens related to deaths and their sensitivity pattern.

**Results:**

In a total of 401 diarrheal children with bacteraemia, 45 (11%) died. Although *Salmonella* Typhi (34%) was the most predominant isolate followed by *Staphylococcus* species (16%) and *Pseudomonas* species (9%), children who died more often had *E*. *coli* (OR = 5.69, 95% CI = 2.42–13.39, p = <0.001) and *Klebsiella* bacteraemia (OR = 4.59, 95% CI = 1.84–11.46, p = 0.001) compared to those who survived. However, none of them was significantly associated with deaths in regression analysis when adjusted with other potential confounders. *E*. *coli* was 100% resistant to ampicillin, 41% to gentamicin, and 73% to ceftriaxone and *Klebsiella* species was 96% resistant to ampicillin, 42% to gentamicin, and 62% to ceftriaxone. Study children who died had significantly higher overall resistance pattern shown in World Health Organization (WHO) recommended one of the first line antibiotics in treating childhood sepsis such as ampicillin (80% vs. 50%, p = 0.001) and in second line antibiotic such as ceftriaxone (49% vs. 22%, p = 0.001) compared to the survivors. In logistic regression analysis, after adjusting for potential confounders, we found that clinical sepsis (aOR 3.79, 95% CI 1.60–8.96, p = 0.002), hypoxemia (aOR 4.20, 95% CI 1.74–10.12, p = 0.001), and hyperkalaemia (aOR 2.69, 95% CI 1.05–6.91, p = 0.039) were found to be independent predictors of deaths and receipt of sensitive antibiotic (aOR 0.42, 95% CI 0.18–0.99, p = 0.048) was revealed as the independent protective factor for deaths in this population.

**Conclusion and significance:**

The results of our data suggest that diarrheal children with bacteremia who died more often had gram negative bacteremia compared to those who survived and these pathogens are highly resistant to WHO recommended first line and second line antibiotics. The results further emphasize the critical importance of early identification of important clinical problems such as clinical sepsis, hypoxemia and hyperkalaemia in diarrheal children and treat them with potential sensitive antibiotic(s) in order to reduce bacteremia related mortality in children with diarrhea, especially in resource limited settings.

## Introduction

The World Health Organization (WHO) estimated that 5.6 million children die each year globally before reaching their 5^th^ birth anniversary. Four communicable diseases were responsible for more than half of these deaths, where diarrhea (8% of all deaths) was the second leading cause [1]. Moreover, almost two third of all diarrheal deaths in this population took place in sub-Saharan Africa and South-East Asia [1]. After the year 2000, most of the developing countries have adopted the Millennium Development Goals (MDGs), especially the Goal-4 that aimed to reduce under-five child mortality, and as a result, diarrheal mortality is decreasing by variable degrees based on different geographical areas. Although, most of the developing countries failed to achieve the goal, Bangladesh is one of the few countries who achieved the goal where diarrhea still contributes 6% to a total of 119,000 deaths in children under five years of age [1]. We are now intending to achieve Sustainable Development Goals (SDGs), and SDG-3 specifically targets to reduce under-five mortality to at least by 25 per 1,000 live births. Hence more emphasis should be given for the reduction of diarrhea related under-five deaths in order to achieve SDG-3.

In Bangladesh, the main etiology of childhood diarrhea is rotavirus [2], where antibiotic has no role in treating these children rather to treat concurrent co-morbidity, if any. If a child with diarrhea has concomitant features of systemic illness such as bacteremia, he or she may requires antibiotic therapy. In such condition, clinicians usually use antibiotics empirically. As the mortality is so high in this special group of children, it is important to understand identifying the factors including probable bacterial pathogens associated with deaths and their sensitivity pattern to antibiotics recommended treating them appropriately in order to prevent their fatal outcome. A number of studies done in children hospitalized with diarrhea were found to be associated with deaths compared to those who did not have bacteremia [3–8]. However, there is scarcity of recent published data regarding the bacterial pathogen related to deaths and antibiotic sensitivity pattern among children with diarrhea and bacteremia. Thus, it warrants the identification of clinical and laboratory characteristics associated with deaths in children hospitalized with both diarrhea and bacteremia with a special attention to bacterial pathogens related to deaths and their sensitivity pattern.

## Materials and methods

### Study site

We conducted this study at Dhaka Hospital of the International Centre for Diarrhoeal Disease Research, Bangladesh (icddr,b), Dhaka, Bangladesh which is the world’s largest diarrheal disease hospital. This is a 100% free of cost hospital which serves about 150,000 patients each year [9]. Majority of the hospital patients are aged below five years (>60%) and they get admitted with the complaints of diarrhea with or without complications and/or associated problems. As a paperless hospital, it has an electronic patients’ data base system to record all clinical and laboratory data. The detailed description of the hospital has been provided somewhere else [10]. Adjunct to ‘Dhaka hospital’, icddr,b has a state of art laboratory facility to do all necessary laboratory investigations of the patients. The laboratory maintains the international standard and it is accredited by ISO-15189 (quality) and ISO-15190 (safety).

### Study design

It was a descriptive chart review from the electronic patients’ data base of ‘Dhaka Hospital’ of icddr,b.

### Study population

We sorted all the under-five children who were admitted in the hospital’s in-patients’ wards with diarrhea from June-2014 to May-2017 and had their positive blood cultures on admission. We excluded the children with Coagulase-negative Staphylococci, *Corynebacterium* sp., *Bacillus* sp., *Micrococcus* sp. and *Candida* sp. etc. from the analysis as those were considered to be non-pathogenic or contaminant by treating physician [11–13]. Patients those who died in the hospital (n = 45) were compared with those who survived (n = 356). Performance of blood culture on admission at in-patients’ ward is not a routine practice in this hospital due to its limited resources. The patients were selected for blood culture based on clinical judgments (as for instance- patients having clinical signs and symptoms of enteric fever, or sepsis, or severe pneumonia, or meningitis etc with diarrhea) by the attending physicians. Demographic, clinical (including severe acute malnutrition, pneumonia, clinical sepsis, and ileus) and laboratory parameters (including bacterial pathogens, antibiotic resistance pattern, and acute kidney injury) were compared between the dead and the survivors to determine the potential associated factors for deaths in children with diarrhea and bacteremia.

### Working definitions

Diarrhea was defined as passage of unusually loose or watery stools, usually at least three times in a 24 hours period [14].

Pneumonia was diagnosed following the World Health Organization (WHO) criteria for children under 5 years of age [15].

Clinical sepsis was defined as presence or suspect of any infection characterized by specific signs and/or symptoms, plus any two of the following: 1) hypo- (≤35.0° C) or hyperthermia (≥38.5° C), 2) abnormal white blood cell (WBC) count (>12x 10^9^ ⁄ L or, <4 x 10^9^ ⁄ L or, band and neutrophil ratio ≥0.1), 3) tachycardia (defined as heart rate above the upper normal limit according to age by WHO), 4) tachypnea (defined as respiratory rate above the upper normal limit according to age by WHO), and 5) abnormal cognition [16].

Ileus was defined if a child developed abdominal distension and had hyperactive or sluggish or absent bowel sound and a radiologic evidence of abdominal gas-fluid level during hospitalization [10].

Acute kidney injury was defined if serum creatinine level greater than the upper limit of normal value (normal values: 18–35 mmol/L for Infant, 27–62 mmol/L for child).

Severe acute malnutrition (SAM) was defined following WHO median chart age and sex as WLZ/WHZ <-3 or presence of nutritional edema [17].

### Methods of microbiological cultures and antimicrobial sensitivity

On admission, with all aseptic precautions two ml of fresh venous blood was collected and seeded directly into BacT/ALERT culture bottles and entered into the BacTAlert 3D system situated at microbiology laboratory of icddr,b. Due to resource constraint, only one blood sample for each participant was collected and tested. Antibiotic susceptibility testing was performed by using Disk Diffusion Method as recommended by the Clinical Laboratory Standards Institute (CLSI) [during study period the laboratory followed the available updated editions of CLSI guidelines (CLSI-2014, CLSI-2015, CLSI-2016, CLSI-2017) [18] with commercial antimicrobial discs (Oxoid, Basingstoke, United Kingdom). As per CLSI guidelines, the laboratory measured the zone diameter of antibiotic inhibitory zone, and later prepared the reports as ‘sensitive’, ‘intermediate’ or ‘resistance’ (SIR) according to the zone diameter marked for ‘SIR’ on the guidelines. Due to limited resources, minimum inhibitory concentrations (MICs) were not used to interpret antibiotic resistance according to CLSI guidelines. Culture and sensitivity reports were available by 48–72 hours of sample collection.

### Data analysis

At first paper based case record forms were used to collect patients’necessary demographic, clinical and laboratory data from hospital patients’electronic database system and then it was transcribed in to a the standard statistical softwares, [Statistical Package for Social Sciences (SPSS), Windows (Version 20.0; Chicago, IL). For continuous variables, the student’s t-test (for normally distributed data) or the Mann-Whitney U test (for non-parametric data) was used to compare between groups. For categorical variables, Fisher’s exact test was used when a cell value of 2/2 table was <5, and for all other cases, Chi Square test with Yates correction was used. We have done these tests for the variables shown in Tables 1–5. Finally, multiple logistic regression was performed (shown in Table 6) after correcting for potential confounders to determine the independently associated factors for deaths in children with diarrhea and bacteremia. Factors those were significantly associated in bivariate analysis in Table 1 and Table 4 were included in the regression model in Table 6. We have also adjusted ‘hospital acquired infection’ in our model as clinically it is a potential important confounder, although it was not statistically significant in bivariate analysis shown in Table 4. A probability of <0.05 was considered as statistically significant, however, in Tables 1 and 4 we adjusted the p value using Bonferroni correction to reduce the risk of a type I error when making multiple statistical tests. Strength of associations was determined by odds ratio and their 95% confidence intervals (CI).

**Table 1:** Distribution of bacterial isolates in blood among deaths and survivors.

Bacteria	Total (n = 401) (%)	Death (n = 45) (%)	Survivor (n = 356) (%)	OR (95% CI)	p*
*Salmonella* typhi	138 (34)	3 (7)	135 (38)	0.12 (0.35–0.38)	**<0.001**
*Staphylococcus* species	65 (16)	3 (7)	62 (17)	0.34 (0.10–1.13)	0.103
*Pseudomonas* species	35 (9)	7 (16)	28 (8)	2.16 (0.88–5.27	0.083
*Escherichia coli*	27 (7)	10 (22)	17 (5)	5.69 (2.42–13.39)	**<0.001**
*Klebsiella* species	24 (6)	8 (18)	16 (4)	4.59 (1.84–11.46)	**0.001**
*Acinetobacter* species	22 (5)	2 (4)	20 (6)	0.78 (0.18–3.46)	1.000
*Streptococcus* species	22 (5)	1 (2)	21 (5)	0.36 (0.05–2.76)	0.491
*Salmonella* paratyphi	9 (2)	0 (0)	9 (2)	-	-
*Campylobacter* species	9 (2)	0 (0)	9 (2)	-	-
Non-typhoidal *salmonella*	8 (2)	1 (2)	7 (2)	1.13 (0.14–9.43)	1.000
*Enterococcus* species	8 (2)	3 (7)	5 (1)	5.01 (1.16–21.74)	0.049
*Shigella* species	7 (2)	1 (2)	6 (2)	1.32 (0.16–11.27)	0.568
Others (combindly) (e.g. *moraxella*, *enterobacter*, *serratia*, *haemophilus parainfluenza*, MRSA etc.)	27 (7)	6 (13)	21 (6)	2.45 (0.93–6.45)	0.118

OR = odds ratio; CI = confident intervals; Chi Square test with Yates correction and Fisher’s exact test was used when a cell value of 2/2 table was <5 was used to compare groups

* Statistically significant p value was considered as <0.003 (0.05/13 = 0.003) after Bonferroni correction.

**Table 2:** Overall resistance pattern of different antibiotics among deaths and survivors.

Antibiotics	Death (n = 45) (%)	Survivor (n = 356) (%)	OR	95% CI	p
Ampicillin	27/34 (79)	141/284 (50)	3.91	1.65–9.27	**0.002**
Gentamicin	13/34 (38)	52/165 (31)	1.34	0.62–2.89	0.448
Ciprofloxacin	16/39 (41)	86/332 (26)	1.99	1.00–3.94	0.070
Azithromycin	17/28 (61)	86/256 (34)	3.05	1.37–6.81	**0.008**
Ceftriaxone	18/37 (49)	70/311 (22)	3.26	1.62–6.55	**0.001**
Cefixime	14/23 (61)	43/210 (20)	6.04	2.45–14.89	**<0.001**
Ceftazidime	11/26 (42)	27/79 (34)	1.41	0.57–3.50	0.607
Amikacin	6/26 (25)	11/89 (12)	2.12	0.70–6.45	0.298
Levofloxacin	4/5 (80)	1/23 (4)			**0.001**

OR = odds ratio; CI = confident intervals; Chi Square test with Yates correction and Fisher’s exact test was used when a cell value of 2/2 table was <5 was used to compare groups.

**Table 3:** Organism specific antibiotics resistance pattern.

Bacterial isolates	Total n (%)	Resistance of antibiotics n (%)								
		AMX	GEN	CIP	AZM	CRO	CFM	CAZ	AMK	LVX
*Salmonella* typhi	138 (34)	36/138 (26)	NS	3/132 (2)	11/138 (8)	0/138 (0)	0/138 (0)	-	-	1/1 (100)
*Staphylococcus* species	65 (16)	55/61 (90)	26/64 (41)	37//62 (60)	10/14 (71)	32/55 (58)	4/5 (80)	1/3 (33)	1/3 (33)	0/2 (0)
*Pseudomonas* species	35 (9)	NS	10/34 (29)	6/35 (17)	-	0/2 (0)	-	4/34 (12)	7/35 (20)	-
*Escherichia coli*	27 (7)	27/27 (100)	11/27 (41)	17/27 (63)	22/26 (85)	19/26 (73)	21/25 (84)	15/24 (62)	4/26 (15)	1/2 (50)
*Klebsiella* species	24 (6)	NS	10/14 (42)	7/22 (32)	18/21 (86)	15/24 (62)	16/21 (76	13/21 (62)	3/21 (14)	1/1 (100)
*Acinetobacter* species	22 (5)	NS	3/22 (14)	8/22 (36)	15/19 (79)	8/22 (36)	13/20 (65)	4/15 (27)	2/21 (9)	-
*Streptococcus* species	22 (5)	3/7 (43)	NS	1/5 (20)	9/21 (43)	3/22 (14)	-	0/2 (0)	0/2 (0)	0/19 (0)
*Salmonella* paratyphi	9 (2)	0/9 (0)	NS	0/9 (0)	2/9 (22)	0/9 (0)	-	-	-	
*Campylobacter* species	9 (2)	4/8 (50)	1/1 (100)	6/8 (75)	3/6 (50)	0/1 (0)	1/1 (100)	0/1 (0)	0/1 (0)	-
NTS	8 (2)	1/8 (12)	NS	0/8 (0)	0/8 (0)	0/8 (0)	0/8 (0)	-	-	-
*Enterococcus* species	8 (2)	6/9 (67)	2/7 (29)	5/8 (62)	2/2 (100)	6/8 (75)	-	1/1 (100)	-	2/2 (100)
*Shigella* species	7 (2)	2/7 (29)	NS	3/7 (43)	4/7 (57)	0/7 (0)	0/1 (0)	-	0/1	-

NS = non-susceptible, AMX = ampicillin, GEN = gentamycin, CIP = ciprofloxacin; CRO = ceftriaxone, CAZ = ceftazidime, AMK = amikacin, AZM = azithromycin; CFM = cefixime; LVX = levofloxacin; NTS = Non-typhoidal salmonella

**Table 4:** Socio-demographic, clinical and laboratory characteristics among deaths and survivors.

Variables	Death (n = 45) (%)	Survivor (n = 356) (%)	OR	95% CI	p*
Age in months (median, IQR)	5.5 (2.8, 10.9)	11.3 (5.5, 27.4)	-	-	<**0.001**
Co-morbidity: Pneumonia on admission	32/44 (73)	100 (28)	6.75	3.34–13.62	**<0.001**
Co-morbidity: Clinical sepsis on admission	32/44 (73)	74 (21)	10.16	4.99–20.69	**<0.001**
Co-morbidity: Ileus on admission	8/44 (18)	43 (12)	1.62	0.70–3.71	0.365
Co-morbidity: Hypoxaemia (SpO_2_ <90%) on admission	29 (64)	39 (11)	14.73	7.35–29.52	**<0.001**
Co-morbidity: Convulsion on admission	10 (22)	29 (8)	3.22	1.44–7.16	0.006
Co-morbidity: SAM on admission	33 (73)	170 (48)	2.97	1.49–5.95	0.002
Dehydration on admission	13 (29)	94 (26)	1.13	0.57–2.25	0.860
H/O fever on admission	28 (62)	265 (74)	0.56	0.29–1.08	0.118
Hospital acquired infection on admission	2 (4)	11 (3)	1.45	0.31–6.80	0.630
Duration of diarrhoea (median, IQR)	4.0 (2.0, 5.7)	4.0 (3.0, 7.0)	-	-	0.607
Duration of fever (median, IQR)	4.0 (3.0, 7.25)	5.0 (3.0, 7.0)	-	-	0.678
Duration of cough (median, IQR)	4.0 (2.0, 5.0)	3.0 (2.0, 6.0)	-	-	0.931
Receipt of sensitive antibiotics	26 (58)	284 (80)	0.35	0.18–0.66	**0.001**
After days sensitive antibiotics received (median, IQR)	1.0 (1.0, 1.0)	1.0 (1.0, 1.0)	-	-	0.735
Abnormal WBC (>11000 or <4000 / cmm) on admission	33/42 (78)	209/348 (60)	2.39	1.10–5.14	0.035
Hypoglycemia on admission	4/28 (14)	5/104 (5)	3.30	0.82–13.22	0.095
Haemoglobin (gm/dl) (mean ±SD)	10.30 ± 2.22	10.34 ±2.01	-	-	0.910
Platelet (per cmm) (median, IQR) on admission	194500 (64000, 480250)	222500 (120000, 405750)	-	-	0.422
Hypernatraemia (Na^+^ >150 mmol/l) on admission	10 (22)	33/284 (12)	2.17	0.98–4.79	0.085
Hyperkalaemia (K^+^>5.3 mmol /L)	15 (33)	26/284 (9)	4.96	2.37–10.39	**<0.001**
AKI on admission	20/44 (45)	63/250 (25)	2.47	1.28–4.78	0.010
Serum Calcium (mmol/l) on admission	2.10 ±0.50	2.16 ± 0.30			0.367
High Mg level (>1.05 mmol/l) on admission	20/38 (53)	28/98 (28)	2.78	1.28–6.02	0.015
Stool culture: organism isolated	6/24 (25)	58/226 (26)	0.86	0.32–2.27	0.950
Time (in hour) to death from admission (median, IQR)	43 (17, 172)	-	-	-	**-**

OR: Odds ratio; CI: confidence interval; SAM: Severe acute malnutrition; H/O: History of; ABx: Antibiotics; IQR: Inter quartile range; WBC: White blood cell; HB: Haemoglobin; SD: Standard deviation; AKI: Acute kidney injury; Mg: magnesium; Hypoglycemia: random blood sugar > 3.5 mmol/L. For categorical variables Chi Square test with Yates correction and Fisher’s exact test was used when a cell value of 2/2 table was <5 was used to compare groups. For continuous variables, the student’s t-test (for normally distributed data) or the Mann-Whitney U test (for non-parametric data) was used to compare groups

* Statistically significant p value was considered as <0.002 (0.05/26 = 0.002) after Bonferroni correction.

**Table 5:** Distribution of stool isolates among deaths and survivors.

Bacteria	Death n = 24 (%)	Survivor n = 223 (%)	OR (95% CI)	p
No growth	18 (75)	168 (75)	0.98 (0.37–2.59)	0.831
*Aromonous caviae*	0 (0)	3 (1)	-	-
*Campylobacter* species	2 (8)	29 (13)	0.61 (0.13–2.72)	0.739
Non typhoidal *Salmonella*	2 (8)	6 (3)	3.29 (0.62–17.28)	0.380
*Salmonella* typhi	0 (0)	5 (2)	-	-
*Shigella boydi*	0 (0)	4 (2)	-	-
*Shigella dysenteriae*	0 (0)	1 (0)	-	-
*Shigella flexnerii*	2 (8)	6 (3)	3.29 (0.62–17.28)	0.380
*Shigella sonnei*	0 (0)	1 (0)	-	-
*Vibrio cholera*	0 (0)	3 (1)		-

OR = odds ratio; CI = confident intervals; Chi Square test with Yates correction and Fisher’s exact test was used when a cell value of 2/2 table was <5 was used to compare groups

**Table 6:** Multiple logistic regression analysis to see the independent predictors of death among the children with diarrhoea and bacteraemia.

Variables	OR	95% CI	P
Age in months	1.02	0.98–1.06	0.232
Pneumonia	2.18	0.90–5.26	0.082
Sepsis	3.79	1.60–8.96	**0.002**
Hypoxemia	4.20	1.74–10.12	**0.001**
Hospital acquired infection	0.59	0.08–4.40	0.595
Receipt of sensitive antibiotics	0.42	0.18–0.99	**0.048**
Hyperkalemia	2.69	1.05–6.91	**0.039**
*Salmonella* typhi	0.65	0.13–3.15	0.599
*Escherichia coli*	1.35	0.45–4.03	0.582
*Klebsiella* species	2.52	0.8–8.09	0.121

OR: Odds ratio; CI: confidence interval; SAM: Severe acute malnutrition; WBC: White blood cell; AKI: Acute kidney injury

### Ethical statement

According to icddr,b’s policy, no permission from the ‘Ethical Review Board’ is required for such retrospective chart analysis as it was not involved with any interview or prospective data collection; however, permission from the Ethical Review Committee (ERC) of icddr,b was obtained to collect data and for analysis. All data were analyzed anonymously and no information of study participants was disclosed.

## Results

**Fig 1:**
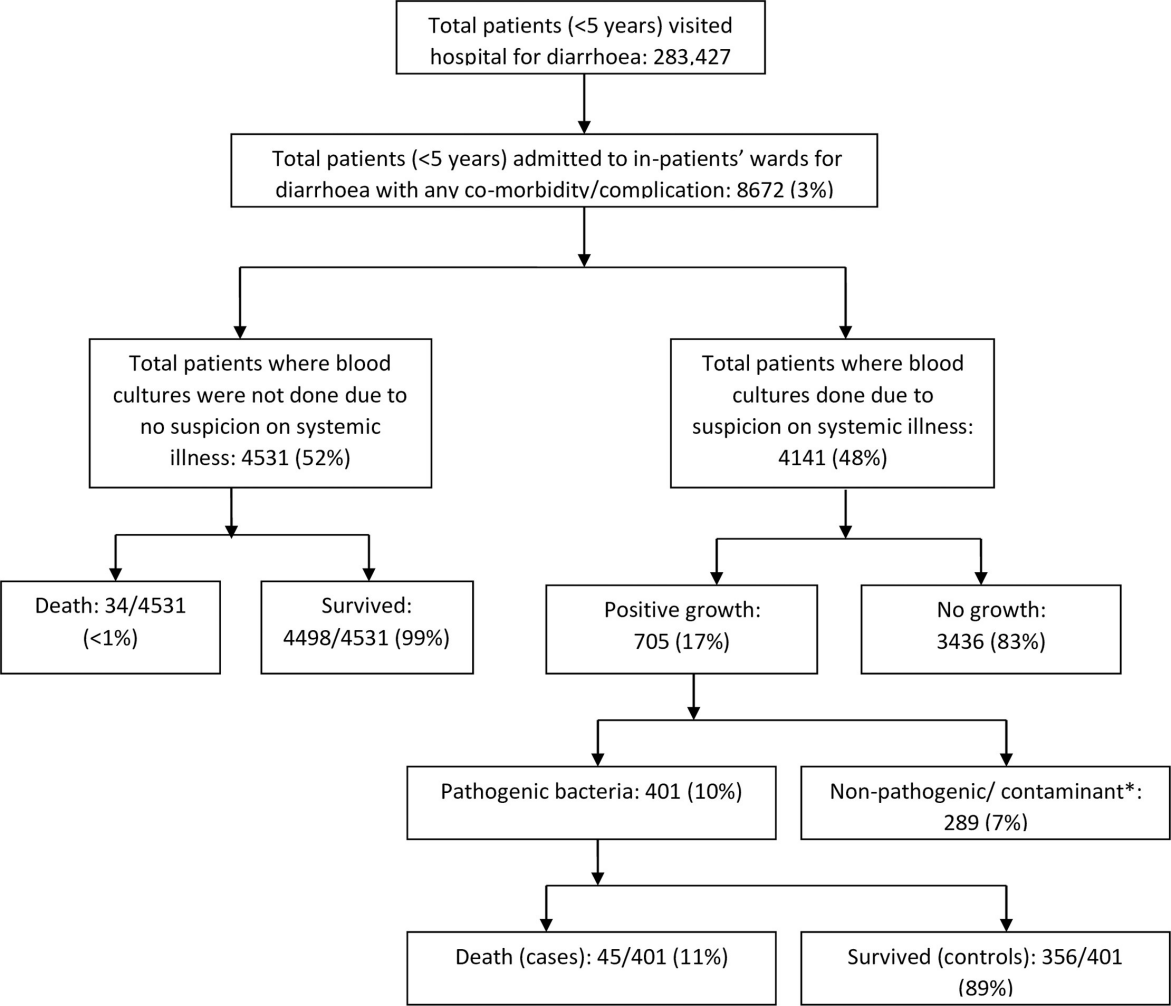
Study flow diagram. * Coagulase-negative staphylococci, *Corynebacterium* spp., *Bacillus* spp, *Micrococcus Spp*. and Candida species.

During the 36 months study period a total of 283,427 under-five children visited this hospital for treatment of diarrhea with/without complication or co-morbidity. Out of them 8,672 patients who were admitted to in-patients’ wards due to diarrhea with any complication/co-morbidity like severe malnutrition, pneumonia, dyselectrolytaemia, persistant diarrhea, typhoid fever etc., 4141 were found to have at least one blood culture done on admission. Out of all the patients who had been at least one blood culture test done, 705 (17%) had bacterial growth. Finally 401 (10%) patients with bacterial isolates were considered for our analysis after deducting 289 cases of probable contaminant or fungi such as Coagulase-negative *Staphylococci*, *Corynebacterium* sp., *Bacillus* sp., *Micrococcus* sp. and *Candida* sp. Isolation rate was 7% (220/2761) in <12 months of age group, and 13% (181/1380) in 12–59 months age group. Out of these 401 enrolled patients, 45 (11%) patients died at hospital (considered as cases) and rest 356 (89%) survived (considered as controls). (Fig 1). Among a total of 4531 children in whom we did not have blood culture, 34 died (<0.75%).

A total of 287/401 (71%) patients had gram negative bacteria. *Salmonella* Typhi (34%) was the most common isolates in this study population, followed by *Staphylococcus* species (16%) and *Pseudomonas* species (9%). However, *Escherichia coli* bacteraemia (22% vs. 5%, p = <0.001) and *Klebsiella* bacteraemia (18% vs. 4%, p = 0.001) were significantly higher among the cases compared to the controls (Table 1); eventually in multiple logistic regression analysis *E*. coli and *Klebsiella* bacteraemia did not remain significant (Table 6).

Overall resistance to commonly used antibiotics (ampicillin, azithromycin, ceftriaxone, cefixime and levofloxacin) against bacterial pathogens (after combining the resistance pattern of all isolates for a specific antibiotics) in the cases was significantly higher compared to the controls (all p = <0.05) (Table 2).

Regarding organism specific antibiotic sensitivity *Salmonella* Typhi had sensitivity ranged from about 90 to 100% against commonly used or empirical antibiotics like ciprofloxacin, ceftriaxone, cefixime and azithromycin shown in Table 3. However, the other gram negative bacteria such as *E*. coli and *Klebsiella* species showed 15% to 100% and 14% to 100% resistance respectively against most of the commonly used/ empirical antibiotics (Table 3). *Staphylococcus* also showed 33% to 90% resistance against these common antibiotics (Table 3).

The cases were more likely to be younger infants compared to the controls (median age 5.5, IQR: 2.8, 10.9 vs. 11.3 months, IQR: 5.5, 27.4; p = <0.001). Median time for deaths from hospital admission was 43 (IQR: 17, 172) hours. Majority (57%) of the patients died within 72 hours of hospital admission. In the bi-variate analysis, the cases more frequently presented with pneumonia, sepsis, and hypoxemia (Table 4). The cases less often received susceptible antibiotics (which was revealed when the blood culture report was available) than the survivors during hospital stay (Table 4). In laboratory investigations, it was found that more patients in the cases had hyperkalemia than the controls. Other variables shown in the table were comparable between the groups (Table 4).

Bacterial isolates in stool were 25% (6/24) and 26% (58/223) respectively in deaths and survivors (Table 4). *Campylobacter* species were most common isolates followed by few non-typhoidal *Salmonella* and *Shigella flexnerii* (Table 5).

In multivariable logistic regression analysis after adjusting with potential confounders, sepsis, hypoxemia, and hyperkalaemia were revealed as the independent predictors of deaths whereas receipt of sensitive antibiotic was found to be independent protective factor for deaths (Table 6).

## Discussion

The most important observation of this study is the predominance in isolation of gram negative bacteria from the blood irrespective of status of associated co-morbidities such as pneumonia or malnutrition. This might be due to breaches of intestinal wall in diarrheal children that may allow translocation of this type of entero-invasive bacteria from the vulnerable gut to systematic infection. However, in this study, we could isolate bacteria in stool samples in about 25% cases only of tested stool samples, both in dead and survivors. It is important to note that stool and blood pathogens were different except for a few cases. Our findings thus fail to decipher the exact cause of the bacteremia, whether it is due to the translocation of bacteria from the gut flora or infiltration in blood of bacteria causing diarrhea.

Importantly, the mortality rate was shown to be significantly higher in *E*. coli and *Klebsiella* bacteraemia, however, in multiple logistic regression analysis *E*. coli and *Klebsiella* bacteraemia became insignificant, may be due to confounding impact of other variables shown in Table 6. In our study, due to lack of resources, we could not perform the status of ESBL resistance, however, it has been showed that these two bacteria had higher resistance levels against most of the commonly used antibiotics including the third generation cephalosporin.

The overall resistances to commonly used antibiotics (ampicillin, azithromycin, ceftriaxone, cefixime and levofloxacin) were higher when we combined the resistance pattern of all isolates for a specific antibiotic. The odds of death were significantly higher where the resistance of these empirical used antibiotics was also higher. In our study, although every child received empirical antibiotics as per hospital management guidelines since admission, a total of 91 (23%) children did not receive sensitive antibiotics during the hospital stay, or before death as the laboratory reports were available after death of the patients due to the fact that blood culture report was available after 72 hours of samples collection. Most of the children initially presented with severe malnutrition, and/or severe pneumonia, and/or suspected typhoid, and/or sepsis etc. as a co-morbidity with diarrhea. For severe malnutrition, the hospital followed the standard management guideline derived from an evidence based treatment guideline, where intravenous ampicillin and gentamycin was the choice of first line antibiotics [19]. Pneumonia was managed as per WHO guideline where the choice of first line treatment was also the same [17]. In case of suspected enteric fever, the children received intravenous ceftriaxone from the first day of admission. Standard practice for changing antibiotics follows available blood culture with sensitivity report or significant clinical deterioration before the availability of the blood culture report. In most of the community acquired infections (except typhoid fever), all our study patients received first line antibiotics (ampicillin + gentamicin). Antibiotics were changed to second line (ceftriaxone + levofloxacin) or (ceftriaxone+ gentamycin) based on ‘no improvement at least after 48 hours’ or ‘clinical deterioration within 24 hours ‘ of initiation of treatment, or on the basis of blood culture report (usually after 72 hours). The children those who died, most of them died before the availability of the blood culture report due to rapid progression of disease and did not receive sensitive antibiotics. However, if blood culture report was available, antibiotics were changed according to the sensitivity report if required. In the death group, comparatively higher percentage of children (42%) did not receive sensitive antibiotic as per their bacterial sensitivity report compared to survivors (20%). Lack of receipt of sensitive antibiotics by 42% children might also contribute for higher numbers of death in children.

Interestingly, among all the isolates, *Salmonella* Typhi was the predominant isolate as an enteric pathogen in blood in our study population. However, death rate was significantly lower in children having *S*. Typhi bacteremia probably due to its marked sensitivity to commonly used or empirical antibiotics like ciprofloxacin, ceftriaxone, cefixime and azithromycin. Although a recent publication from Nepal showed most isolates (86%) of *S*. Typhi were non-susceptible to fluoroquinolones [20], in our population it was intermediate sensitive and as per hospital management guideline all patients with *S*. Typhi received ceftriaxone.

In case of diarrhea with co-morbidity or systemic illness, it would be difficult to predict the actual causative organism clinically. Thus, the clinicians in resource poor settings usually prefer to prescribe one or more empirical antibiotics having relative wide coverage and evidence of good sensitivity following updated local surveillance of causative organism and their antibiotic sensitivity.

This study reported that the isolation rate of pathogenic bacteria in blood was about 10% in under-five children with diarrhea having presumed sepsis or other co-morbid conditions (n = 4141) on admission. In this study, the selection of cases for performing blood culture was under jurisdiction of the clinician and thus subjective to selection bias. Henceforth, those findings may not reflect the prevalence of bacteremia in this population; rather it would be the average picture in the diarrheal children with common co-morbidities. The death rate in this study in bacteremic children having diarrhea was about 11%, which was lower than a number of previous studies conducted in Bangladesh (case fatality rate: 30–33%) [3–5]. This might be due the fact that all these previous studies included children with clinical syndrome of sepsis or other serious co-morbidities. Other studies in Asia and Africa reported that the case fatality for community acquired bacteraemia varied from 16%-38% in children with malaria, or HIV or illness other than diarrhea [21–23].

The observation of clinical sepsis, hypoxemia and hyperkalaemia as the independent predictors for death in children with diarrhea and bacteremia is understandable. Sepsis is a clinical syndrome resulting from a dysregulated systemic inflammatory response to infection and characterized by a generalized pro-inflammatory cascade, which may lead to widespread tissue injury [24]. So, often it leads to severe sepsis, septic shock and multi-organ failure [25] where the death rate is very high in both developed and developing countries [26, 27]. The mortalities vary from 4–34% in sepsis to septic shock [28–30]. An earlier study in Bangladesh showed the mortality was about 15% in this age group (under five) [27].

Hypoxemia is another most detrimental clinical consequence for children with severe illness like pneumonia [31, 32]. It results from alveolar consolidation which causes marked reduction in gases exchanges. Death rate varies from 8–22% in hypoxemic children in different geographical locations [32, 33]. In our study, hypoxemia was found as a predictor of death in diarrhea and bacteremia.

Although in acute diarrhea patients may excrete potassium through stool, still they may develop hyperkalemia due to infection cascade. This intracellular substance can be increased in blood due to reduced renal excretion or leakage of potassium from the intracellular space [34]. Patients with dehydrating diarrhea or sepsis often present with metabolic acidosis and this may cause intracellular potassium to shift into the extracellular space [35]. Patients with severe hyperkalemia may end up with fatal outcome [36].

Our observation of receipt of sensitive antibiotic as the independent protective factor for deaths is really important. Our observation is consistent with a number of previous studies [37, 38]. This observation in our study underscores the importance of early choice and administration of potential susceptible antimicrobials in children with bacteremia that may help to reduce the mortality in such children especially in resource poor settings.

There are some limitations in this study. As the data were obtained from a resource limited setting, we could not perform blood culture of all admitted children with diarrhea in the hospital, rather the blood culture was performed as per the clinical judgment of the attending physician. Hence we are unable to determine the actual rate of bacteremia in all the children having diarrhea. We did not have information on the use of antibiotics prior to admission over the counter or from outpatient healthcare providers since these may have an impact in skewing the blood culture susceptibility findings. We did not have data of excluded non-pathogenic patients that might provide more information. Due to retrospective nature of the study, socio-demographic variables were very limited and specific information on the number of children received first line antibiotics and switched to second line antibiotics is not available. Minimum inhibitory concentration (MIC) and sero-typing of many bacterial isolates could not be done due to financial constraint involved with the study.

In conclusion, the results of our data suggest that gram negative bacteria were common in diarrheal children those who developed bacteremia. Stool cultures do not predict organisms isolated in the blood, thus the importance of blood cultures in the management of these complicated cases are imperative. The study also noted that one out of ten children under five died of diarrhea with bacteremia. *Escherichia coli* and *Klebsiella* species were multidrug resistance pathogens. Clinical sepsis, hypoxemia and hyperkalemia were independently associated factors for bacteremic deaths. However, use of organism specific sensitive antibiotics revealed as the protective factor for death in this population. Therefore, early identification of sepsis, hypoxemia and hyperkalemia and treatment with potential sensitive antibiotic(s) based on local antibiotics sensitivity profile of the common isolates is imperative in order to prevent the mortality in diarrheal children with bacteremia. Further study to evaluate the current antibiotic resistance pattern against common causative organism may play important role to update the treatment guidelines with sensitive antibiotics.
